# Exploring the medicine cost of managing Diabetes Mellitus in Nepal: A cross-sectional analysis of oral hypoglycaemic medications

**DOI:** 10.1371/journal.pone.0310706

**Published:** 2024-12-05

**Authors:** Rajeev Shrestha, Amrita Aryal, Asmita Priyadarshani Khatiwada, Shreya Dhungana, Sunil Shrestha

**Affiliations:** 1 Palliative Care and Chronic Disease, INF Green Pastures Hospital and Rehabilitation Centre, Pokhara, Province Gandaki, Nepal; 2 Department of Pharmacy, School of Medical Sciences, Kathmandu, Province Bagmati, Nepal; 3 Department of Pharmacy, National Model College for Advanced Learning, Kathmandu, Province Bagmati, Nepal; 4 Department of Research and Academics, Kathmandu Cancer Centre, Tathali, Bhaktapur, Province Bagmati, Nepal; District Head Quarter (DHQ) Hospital Charsadda / University of Peshawar, PAKISTAN

## Abstract

Diabetes Mellitus is a significant global public health burden. Although medication adherence is an inevitable consideration in managing and curing the disease, medication price is the major barrier to patients in Nepal, like any other low- and middle-income countries. Prescribing in brand-name inhibits the possibility of accessing cost-effective generic alternatives in Nepal. This study aimed to explore and examine price variations among the oral hypoglycaemic medicines (OHMs) available in the country and their distribution in various medicine-related characteristics. A cross-sectional study was conducted using a convenient sample of five tertiary care hospital pharmacies in Kathmandu, publicly accessible online pharmacy websites, and a government database to list the OHMs available in Nepal. The study determined price variations and statistically tested the association between these variations and the characteristics of the medicines. Fourteen OHMs were available as 57 generic medicine items with different formulations in Nepal. The maximum of 484.82% of price variation was found. Fourteen, fifteen and eighteen OHMs have variations of over 100%, more than 10 rupees and no price change, respectively. Except for Dipeptidyl Peptidase-IV (DPP-4) Inhibitors and Thiazolidinediones (TZDs), all other categories of OHMs have >100% of price variation in medicine items. Although Nepal itself produces most of the available OHMs, the available OHMs have price variations. Most fixed dose combinations showed no reduction in cost compared to their component medicine’s mean price. This study presented and discussed the price variation scenario of OHMs with their medicine-related characteristics to develop and implement effective drug policies and programs that can address medication price-related issues to ensure access to OHMs without placing an economic burden on patients.

## Introduction

Diabetes Mellitus (DM) is a significant global public health burden, reported to have caused 1.5 million global deaths in 2019 [1]. The global prevalence of DM was estimated at 9.5% in 2019 and is expected to rise up to 10.2% in 2030 and 10.9% in 2045 [2]. Subsequently, the prevalence of diabetes and pre-diabetes was reported as8.5% and 9.2% in 2020 in Nepal, respectively [3, 4]. Notably, DM incidence is rising quickly in low-and middle-income countries (LMICs), including Nepal [1, 5–7]. This has significantly affected the clinical outcomes and economic conditions of patients and the nation [8]. Yang et al. (2019) reported that individuals with diabetes have a 1.89-fold greater risk of death than non-diabetes in Asia [9]. Similarly, hypertension, a prevalent issue in Nepal, has double the risk of developing diabetes in Nepal [4, 10]. As per Shrestha et al. (2013), patients with DM for 16 to 20 years have 161% higher direct health expenses compared to those with DM for 1 to 5 years [11]. Overall, type 2 DM (T2DM) complications cost up to nine times higher than non-T2DM patients [12].

Therefore, good adherence to medication can prevent further complications, mortality and associated economic burden which might occur in time to come [13–15]. A study found DM patients spend up to 80% of their total expenses on medications during out-patient clinic visits in Nepal, primarily through out-of-pocket (OOP) payments [11]. In Nepal, a substantial portion of health expenditure is dedicated to medicine and medicinal products, accounting for 63.4% of out-of-pocket (OOP) expenses. Non-communicable diseases (NCDs) make up two-thirds of these total OOP expenses on diseases [16]. Similarly, past studies have reported finances (participants’ income and medicine costs) as a significant challenge to medication adherence for DM patients in Nepal [17, 18]. Furthermore, the high price variation of NCD medications, including oral hypoglycaemic medicines (OHMs), reported earlier, has further added barriers to medication adherence in DM patients in Nepal [19–21].

Most medicines available in Nepal are brand-named generics, meaning they have the same active ingredient but different trade names. According to the Department of Drug Administration (DDA), there are 128 pharmaceutical manufacturers in Nepal as of 29^th^ September 2022, with118 producing allopathic medicines, and 123 registered importers according to online data as of 19th October 2023 [22, 23]. However, Nepal lacks specific regulations in medicine pricing and controlling price variation between brand-named medicines of the same generic medicine items [24, 25]. Despite this, the government attempted to ensure access by fixing the price of some essential medicines, providing some essential medicines for free in limited centres, and including a broader range of medicines (not all OHMs) in the national health insurance scheme [26–28]. However, these freely available medicines, including OHMs, are inadequate and are reported to be of substandard quality [29, 30]. Moreover, health insurance services are also available in limited facilities, and patients have been reported to withdraw from this scheme due to inadequate medicine availability [31, 32]. These factors force patients to depend on OOP expenditure and private pharmacy outlets, facing high and inconsistent medicine costs.

All the above-mentioned medicine-related characteristics, including formulation, composition, manufacturer competition, dosage form types, and coverage in the national medicine program list, can influence price of the medicine and its price variation among different manufacturers [33–36]. However, none of the previously published studies have examined the detailed analysis of the availability and variation in the prices of available OHMs in the country [20, 21, 37–39]. Thus, this study aimed to investigate the price variation of OHMs based on different medicine-related characteristics to provide a comprehensive overview and suggest better ways to manage medicine costs in the future.

## Materials and methods

### Study design

A cross-sectional study was designed to examine the price variation of OHMs available in Nepal and was conducted from April 2023 to September 2023.

### Ethical approval

Ethical approval for this study was obtained from the Ethical Review Board of Nepal Health Research Council (NHRC) in Kathmandu, Nepal (Reference Number: 51). Pharmacy representatives were informed about the study in detail and data were collected after receiving their written and verbal consent.

### Sampling and data collection

Initially, ten pharmacies from five tertiary care hospitals in Kathmandu, the capital city of Nepal (two pharmacies from each hospital), were conveniently selected for the market survey to ensure overall coverage of oral hypoglycaemic medicines available in Nepal. The survey collected information such as brand name, generic name, strength, dosage form, maximum retail price, manufacturer name, and country. Two pharmacists with previous work experience in a hospital pharmacy setting conducted the market survey. They prepared the final list of OHMs and cross-checked the publicly available online pharmacy shops and the Department of Drug Administration (DDA) database to avoid the possibility of missing any OHMs available in the Nepalese market [40–42]. If any medicine information was missed, we contacted the pharmacy representatives and utilized their network to obtain the missing information. This process of double-checking further ensured the genuineness of the medicine details obtained from the selected hospital pharmacies.

The study concentrated on five tertiary care hospitals in Kathmandu, a capital city of Nepal These hospitals were chosen for their higher level of care and their role as referral centres for diabetic patients. Hospitals in Kathmandu were chosen as they offer the highest possible level of care in Nepal, thus expected to have access to all OHMs available in Nepal. The researchers chose online pharmacy shops and the DDA online database to verify medicine availability due to its convenience, time-saving nature, and widespread use in urban areas of Nepal. In addition, to remove the possibility of doubt about variations in medicine prices at virtual and physical pharmacies, we cross-checked the medicine maximum retail price (MRP) from retail pharmacies before making a final data analysis. The rate was collected at the same place and at a specified time (1^st^ to 7^th^ August 2023), preventing the possibility of rate differences due to different batches of the same brand-named medicines with different rates at various sites and times of data collection. All the MRPs are presented in the Nepalese Rupee (NPR).

### Study variables

The dependent variable of this study was price variation. The independent variables were medicine-related characteristics. They are the number of active ingredients in each generic medicine item, the number of brand-named medicines in each generic medicine item, number of generic medicine items from government’s fixed rate medicine list, number of generic medicine items from WHO Essential Medicine List (EML), number of generic medicine items from Nepal Essential Medicine List (EML), number of generic medicine items from the government free medicine list of Nepal, number of generic medicine items from Health Insurance Medicine list of Nepal, number of types of dosage forms of OHMs, and number of manufacturing countries for each generic medicine item.

### Data analysis

The price presented in the result section refers to the MRP of the individual product’s specific strength and dosage form. The study calculated the mean, median, interquartile range, minimum, maximum, and percentage price variation for each product to visualise the price variation of different brand-name medicines of the same generic medicine item with similar strength and dosage form. The formula used for calculating price variation in percentage was: price variation in percentage = [(Price of brand-named medicines having highest MRP–Price of brand-named medicines having lowest MRP)/ price of brand-named medicines having lowest MRP]*100. Furthermore, OHMs were categorized based on their pharmacological effect, and their price distribution was analysed [43]. A comparison of the price of fixed-dose combination (FDC) and individual drug dosage was also reviewed and studied wherever possible. The study evaluated the price variation between FDCs and the individual prices of combined medicines. The formula used was Percentage price variation = [(mean price of FDCs—mean price of individual combined medicines) / mean price of individual combined medicines] x 100. The researchers also compared the available OHMs with the Nepalese Essential Medicine List (2021), the WHO Essential Medicine List (2021), and the Nepalese Government Free, Price Fixed and Health Insurance Medicine List [26, 44–47].

Before applying the statistical test, the normal distribution of the outcome variable was checked using Kolmogorov-Smirnov and Shapiro-Wilk tests. The test showed non-linearity of price variation variables (P<0.05). Therefore, the Mann-Whitney U-test was used for the variables having two groups, and the Kruskal-Wallis test was applied for the variable having more than two groups while testing the significant distribution of price variation in medicine characteristics.

Data management and analysis were conducted using Microsoft Excel 2010 and IBM Statistical Package for Social Science (SPSS) version 25.0.

### Operational definitions

#### Types of medicines

This refers to specific oral hypoglycaemic medicines having same active pharmaceutical ingredient (API) irrespective of dose and dosage form (e.g., both metformin 500 mg “Immediate Release (IR)” and metformin 1 gm “Modified Release (MR)” were considered one type of medicine).

#### Generic medicine item

This refers to the specific dosage, combination and dosage form of oral hypoglycaemic medicine available (e.g., metformin 500 mg IR, metformin 500mg + glimepiride 1 mg IR).

#### Brand-named medicines

These refer to oral hypoglycaemic medicines with different trade names from their manufacturers but with similar doses and dosage forms (e.g., Metfor 500 mg IR, Netformin 500 mg IR–both have the same molecule, dose and dosage form, metformin 500 mg IR).

## Results

### Characteristics of oral hypoglycaemic medicines available in Nepal

Fourteen oral hypoglycaemic medicines and 57 generic medicines with different doses and dosage formulations are available in Nepal. Among 57 generic medicine items, 17 medicines are available in fixed-dose combinations under two types in terms of pharmacological categories (second generation sulfonylureas + biguanides and dipeptidyl peptidase-IV (DPP-4) inhibitors + biguanides) and 40 medicines are available as single molecules in their respective dosage forms. Among 57 generic medicine items, 48 medicines were “Immediate Release (IR)”, and 9 were “Modified Release (MR)” dosage formulations.

Similarly, those 57 different OHMs were available in 348 trade/brand names in Nepal. There was a maximum of 29 brand-named medicines and a minimum of one brand-named medicine available for each generic hypoglycaemic medicine item in Nepal. A maximum of up to four countries were found to be involved in manufacturing one generic medicine item. Around 90% of medicines available in the Nepalese market were not on the WHO and Nepal’s essential medicines lists. Similarly, among 57 generic medicines items, the government has fixed the rate for only seven items and provided only one medicine free of cost to designated government healthcare institutions (Table 1). All the details of medicines are given in the **S1 File**.

**Table 1:** Characteristics of OHMs available in Nepal and their relationship with price variation [N = 57].

Characteristics	Number (%)	Average Price variation in %	P-value
**Number of Active Pharmaceutical Ingredients (API) in each generic medicine item** ^#^			
One	40 (70.2)	66.15	0.639
Two	17 (29.8)	53.16	
**Number of available brand-named medicines in each generic medicine item** ^##^			
1.0	15 (26.3)	0	0.000**
2.0	6 (10.5)	4.54	
3.0	2 (3.5)	175.46	
4.0	5 (8.8)	31.57	
5.0	2 (3.5)	57.93	
6.0	6 (10.5)	134.91	
7.0	5 (8.8)	157.77	
8.0	2 (3.5)	116.13	
9.0	2 (3.5)	118.87	
10.0	1 (1.8)	16.96	
11.0	2 (3.5)	15.91	
12.0	4 (7)	110.98	
16.0	2 (3.5)	8.3	
17.0	1 (1.8)	6	
22.0	1 (1.8)	222.22	
29.0	1 (1.8)	92.31	
**Number of generic medicine items from government’s fixed rate medicine list** ^#^			
Listed	7 (12.28)	17.14	0.329
Not listed	50 (87.72)	68.60	
**Number of generic medicine items from WHO Essential Medicine List (EML)** ^#^			
Listed	5 (8.8)	62.92	0.720
Not listed	52 (91.2)	62.22	
**Number of generic medicine items from Nepal Essential Medicine List (EML)** ^#^			
Listed	6 (10.5)	129.13	0.059
Not listed	51 (89.5)	54.41	
**Number of generic medicine items from the government free medicine list of Nepal** ^#^			
Listed	1 (1.8)	92.31	0.422
Not listed	56 (98.2)	61.74	
**Number of generic medicine items from Health Insurance Medicine list of Nepal** ^#^			
Listed	27 (47.4)	74.48	0.015*
Not listed	30 (52.6)	51.30	
**Number of types of dosage forms of Oral Hypoglycaemic medicines** ^#^			
Immediate Release (IR)	48 (84.2)	71.83	0.023*
Modified Release (MR)	9 (15.8)	11.36	
**Number of Manufacturing Countries for each generic medicine item** ^##^			
One	30 (52.6)	13.67	0.000**
Two	22 (38.6)	110.83	
Three	2 (3.5)	39.43	
Four	3 (5.3)	207.60	

*indicate significant at P<0.05

**indicate significant at P<0.01

Mann-Whitney U-test^#^ and the Kruskal-Wallis test^##^

Oral Hypoglycaemic medicines available in Nepal: *acarbose*, *empagliflozin*, *glibenclamide*, *gliclazide*, *glimepiride*, *glipizide*, *linagliptin*, *metformin*, *pioglitazone*, *repaglinide*, *rosiglitazone*, *sitagliptin*, *teneligliptin*, *voglibose*

### Relationship between price variation of each generic medicine item and its characteristics

Table 1 presents the assessment of the statistical relationship of price variation with different characteristics of medicines. The test showed a significant association of price variation of each generic medicine item with the number of types of dosage forms, number of brands available under each generic medicine item, number of manufacturing countries, and number of medicines listed in government social security health insurance service. On the other hand, the medicines listed in EML of Nepal, the medicines listed in EML of WHO, the government-free medicine list, and the number of APIs have not shown a significant relationship with the price variation of each generic medicine item.

### Manufacturer details of OHMs available in Nepal

**Fig 1:**
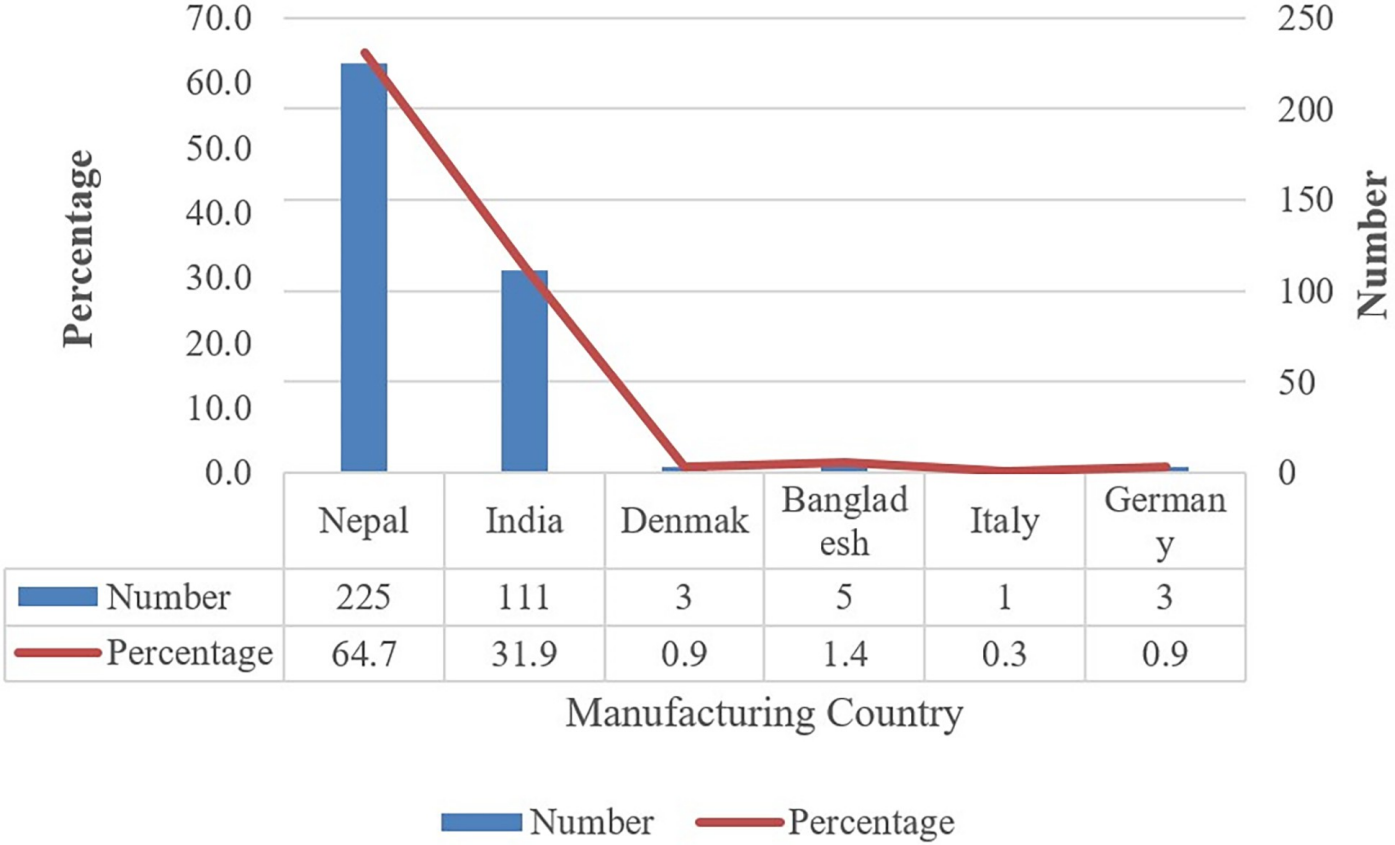
Manufacturer details of OHMs available in Nepal.

Nepal is the prominent manufacturer (64.7%) of OHMs in the Nepalese market, followed by its neighbouring country, India (31.9%). Bangladesh occupied 1.4% of OHMs available in the Nepalese market, while Denmark, Germany and Italy occupied less than 1% of OHMs in the Nepalese market (Fig 1).

### Price variation of oral hypoglycaemic medicines

An average of 62.28% price variation was determined among all OHMs in Nepal. There were three medicines with price variations >200% and eleven with price variations between 100 to 200%. Altogether, fifteen medicines have price differences above 10 NPR with a maximum of 50 NPR. Gliclazide 60 mg MR had the lowest price difference (0.03), while empagliflozin 10 mg IR and 25 mg IR had the highest price difference (50 and 39.52, respectively) (Table 2). However, 18 generic medicine items showed no price variation. None of the government-fixed rate medicines were priced higher than their set rates. Detailed information on the price variations of all the over-the-counter medicines (OHMs) can be found in **S1 File**.

**Table 2:** Top 20 oral hypoglycaemic medicines with the highest price variation available in Nepal.

S.N.	Generic name (dosage form + dose)	No of brands	Minimum price (in NPR.)	Maximum price (in NPR)	Difference in prices (in NPR)	Price variation (%)	Median price	Mean	IQR
1	Repaglinide 2 mg IR	7	4.15	24.27	20.12	484.82	12.00	12.89	10.43
2	Glipizide 5 mg IR	3	0.67	2.50	1.83	273.13	0.96	1.38	0.92
3	Metformin 850 mg IR	22	1.98	6.38	4.40	222.22	3.50	3.45	0.69
4	Linagliptin 2.5 mg + Metformin 1000 mg IR	6	15.40	45.79	30.39	197.34	16.50	21.20	1.00
5	Voglibose 0.2 mg IR	6	5.66	16.45	10.79	190.64	11.00	11.02	0.00
6	Empagliflozin 10 mg IR	8	32.00	82.00	50.00	156.25	37.00	42.25	8.50
7	Glimepiride 1 mg + Metformin 500 mg IR	12	7.00	17.65	10.65	152.14	9.75	10.45	2.71
8	Voglibose 0.3 mg IR	6	9.01	22.00	12.99	144.17	15.00	15.17	0.00
9	Glimepiride 4 mg IR	9	9.68	22.64	12.96	133.88	14.00	15.41	6.90
10	Acarbose 50 mg IR	7	12.00	27.00	15.00	125.00	16.50	17.20	4.45
11	Glimepiride 2 mg + Metformin 500 mg IR	12	10.10	22.40	12.30	121.78	13.00	14.30	2.26
12	Gliclazide 40 mg IR	6	2.80	6.00	3.20	114.29	4.42	4.55	1.96
13	Glimepiride 2 mg + Metformin 1000 mg IR	6	13.00	27.20	14.20	109.23	15.25	17.05	2.96
14	Metformin 500 mg + Sitagliptin 50 mg IR	9	20.00	40.77	20.77	103.85	25.00	25.75	4.00
15	Repaglinide 1 mg IR	4	9.00	17.33	8.33	92.56	12.15	12.66	6.06
16	Metformin 500 mg IR	29	1.04	2.00	0.96	92.31*	2.00	1.89	0.00
17	Sitagliptin 50 mg IR	12	18.60	35.00	16.40	88.17	24.50	23.80	4.25
18	Sitagliptin 100 mg IR	12	27.50	50.00	22.50	81.82	33.50	34.13	5.00
19	Linagliptin 2.5 mg + Metformin 850 mg IR	3	9.00	16.00	7.00	77.78	14.30	13.10	3.50
20	Metformin 850mg MR	8	4.25	7.48	3.23	76.00	4.50	4.90	0.13

Note: 1 US Dollar = 132.85 NPR (Nepalese Rupee) according to the exchange rate of 7^th^ August, 2023; IQR = interquartile range; *price variation is negative meaning the price variation is below government fixed rate (2 NPR)

### Pharmacological category-wise presentation of price variation and medicine summary

Altogether, a total of 7 pharmacological categories of oral hypoglycaemic medicines are found in a total of 14 types of medicines in Nepal. The most common pharmacological class of OHMs was second-generation sulfonylureas (4, 28.57%), followed by DPP-4 inhibitors (3, 21.43%) based on the types of medicines. However, based on the number of brand medicines in the market, biguanides (86, 24.71%) and second-generation sulfonylureas (78, 22.41%) were the most common medicines used in the Nepalese market. Except for dipeptidyl peptidase-IV (DPP-4) inhibitors and thiazolidinediones (TZDs), all other pharmacological categories have more than 100% price variation medicines. Biguanides, 2nd generation sulfonylureas, and meglitinides categories of medicines have an item with more than 200% of price variation. Except for alpha-glucosidase inhibitors and meglitinide categories of medicines, all other categories have medicines with a minimum of zero price variation (Table 3).

**Table 3:** Pharmacological category-wise presentation of price variation and medicine summary.

Pharmacological Categories	Number of types of medicines (%)	Number of generic medicine items (%)	Number of brands (%)	Minimum price variation item (no of brands, price variation %)	Maximum price variation item (no of brands, price variation %)
Second Generation Sulfonylureas	4 (28.57)	14 (24.56)	78 (22.41)	Glibenclamide 2.5 mg IR (1,0), Gliclazide 160 mg IR (1,0), Gliclazide 30 mg IR (1,0), Gliclazide 30 mg MR (1,0), Glimepiride 3mg IR (11,0) Glipizide 2.5 mg IR (1,0)	Glipizide 5 mg IR (3, 273.13)
Second Generation Sulfonylureas+ Biguanides	-	8 (14.04)	40 (11.49)	Glibenclamide 5 mg + Metformin 500 mg MR (1,0), Glimepiride 2 mg + Metformin 850 mg IR (1,0), Glimepiride 3 mg + Metformin-1000 mg IR (1,0), Glimepiride 4 mg + Metformin 1000 mg IR (1,0), Gliclazide 30 mg IR (1,0)	Glimepiride 1 mg + Metformin 500 mg IR, (12, 152.14)
Alpha-Glucosidase Inhibitors	2 (14.29)	4 (7.02)	26 (7.47)	Acarbose 25 mg IR (7,72.86)	Voglibose 0.2 mg IR (6, 190.64)
Biguanides	1 (7.14)	6 (10.53)	86 (24.71)	Metformin 250 mg IR (1,0)	Metformin 850 mg IR (22, 222.22)
Dipeptidyl Peptidase-IV (DPP-4) Inhibitors	3 (21.43)	7 (12.28)	42 (12.07)	Sitagliptin 25 mg IR (4,0), Linagliptin 10 mg IR (1, 0)	Sitagliptin 50 mg IR (12, 88.17)
Dipeptidyl Peptidase-IV (DPP-4) Inhibitors + Biguanides	-	9 (15.79)	42 (12.07)	Metformin 1000 mg + Sitagliptin 50 mg MR (1,0), Metformin 500 mg + Sitagliptin 50 mg MR (2,0), Metformin 850 mg + Sitagliptin 50 mg MR (1,0)	Linagliptin 2.5 mg + Metformin 1000 mg IR (6, 197.34)
Meglitinides	1 (7.14)	3 (5.26)	15 (4.31)	Repaglinide 0.5 mg IR (4,45.43)	Repaglinide 2 mg IR (7, 484.82)
Sodium-Glucose Co-Transporter-2 (SGLT2) Inhibitors	1 (7.14)	3 (5.26)	14 (4.02)	Empagliflozin 5 mg IR (1,0)	Empagliflozin 10 mg IR (8, 156.25)
Thiazolidinediones (Tzds)	2 (14.29)	3 (5.26)	5 (1.44)	Rosiglitazone 4 mg IR (1,0)	Pioglitazone 15 mg IR (2, 3.17)
**Total**	**14 (100)**	**57 (100)**	** 348 (100)**		

Note: 1 US Dollar = 132.85 NPR (Nepalese Rupee) according to the exchange rate of 7^th^ August, 2023

### Comparison of price between fixed-dose combination and their sum of medicines

Out of seventeen FDCs, nine FDCs have a comparatively higher mean price than the sum of their individual item’s mean price, while only 4 FDCs have a lower mean price than the sum of their individual item’s mean prices. Second-generation sulfonylureas + biguanides (glimepiride 1 mg + metformin 500 mg IR and glimepiride 2 mg + metformin 500 mg IR) were found to have high price differences (45.8% and 37.1%, respectively). All other FDCs have less than 25% price variation with their sum of medicines (see **S2 File**).

## Discussion

The study found seven pharmacological categories of oral hypoglycaemic medicines available in Nepal. The most commonly available categories of medicines in the market were second-generation sulfonylureas (4, 28.57%), followed by dipeptidyl peptidase-IV (DPP-4) inhibitors (3, 21.43%). Based on the number of available brand-named medicines, the most frequently used medicines in the market were biguanides (86, 24.71%) and second-generation sulfonylureas (78, 22.41%). These categories of medicines, particularly glimepiride and metformin, are recommended in diabetes management guidelines and are commonly used in Nepal and its neighbouring countries such as India [48–54]. Therefore, the quality and cost of these OHMs must be monitored stringently with priority. The government of Nepal has fixed the price of metformin 500 mg IR and 1000 mg IR, glimepiride 1 mg IR, 2 mg IR, and 3 mg IR and pioglitazone 15 mg IR and 30 mg IR so far [26]. Metformin 500 mg IR is made freely accessible in designated government healthcare centres [47]. Previously, limited studies reported higher price variation on one of the fixed-rate medicines (metformin 500 mg IR, 330.769% in 2015 and 254.6% in 2017) [21, 39]. However, the current study showed lower price variation of government fixed-rate medicines (glimepiride 1 mg IR, 6%, glimepiride 2 mg IR, 7.5%, glimepiride 3, 0%, and metformin 500 mg IR, 92.31%) without crossing the government ceiling price. The possible cause of less price differences despite having fixed retail prices could be the higher market competition (metformin 500 mg IR, 29 brands; glimepiride 1 mg IR, 17 brands, 2 mg IR, 16 brands, and 3mg IR, 11 brands), frequent prescription of metformin and glimepiride in Nepal and involvement of foreign manufacturer [48–50].

Except for dipeptidyl peptidase-IV (DPP-4) inhibitors and Thiazolidinediones (TZDs), all other pharmacological categories of medicines have more than 100% price variation. Biguanides, second-generation sulfonylureas, and meglitinide are medicines with more than 200% price variation. Fourteen generic-medicine items have a price variation greater than 100%, fifteen have a price variation of more than 10 rupees, and eighteen medicines don’t have any price variation. Specifically, repaglinide 2mg IR (484.82%, 7 brands), glipizide 5mg IR (273.13%, 3 brands), and metformin 850mg IR (222.22%, 22 brands) were the highest price variation items found in our study. However, a recent study in India showed glimepiride 1 mg (3503.22%, 108 brands), and metformin 500 mg SR (3668.51%, 73 brands) were the medicines with the highest price variation, which are the most commonly used medicines in Nepal [55]. The price variation of these items in Nepal was much lower compared to India.

Moreover, FDCs are commonly used in the Nepalese pharmaceutical market to reduce drug non-adherence and overall cost reduction [56]. However, the cost comparison showed no reduction in cost by most FDCs; instead, the cost of FDCs was higher compared to the cost of its component medicines. Nine FDCs were found to have higher mean cost than the sum of their components’ mean price, where glimepiride 1 mg + metformin 500 mg IR (45.8%) and glimepiride 2 mg + metformin 500 mg IR (37.1%) were found to have the highest price difference. In our context, the higher prices of FDCs could have occurred using the mean price of all available brand-named medicines. Nevertheless, it is determined that most FDCs’ component generic medicine items can be found at lower prices in the Nepalese market if the patient wants to buy them separately. Still, a question arises among consumers about the higher price of FDCs compared to the sum of their individual components even though the cost of labour, packaging, transportation, and other items born by the manufacturer has already been reduced by almost half.

The study revealed a significant difference in price variation among different number of brand-named medicines available for each generic medicine item. However, the p-value did not provide a conclusive remark adequately due to the limited data (less than 5 brand-named medicines in some generic medicine items). Alternatively, more brand-named medication options for generic medicine items were found to increase. A 2017 Nepalese study reported 1 and 18 brand-named medicines for glibenclamide 5mg and metformin 500 mg [21], which was found to increase in our study, 2 and 29, respectively. This possibly reflects the growth in the availability of the brand alternatives. However, the price differences among the brand-named medicines may direct patients into buying expensive items despite providing the opportunity to choose cost-effective ones. This can be suspected due to the prevalent practice of prescribing in brand names, brand promotion, and patients’ deficient medicinal knowledge and practice of choosing prescriber-prescribed brand-named medicines in Nepal [57–59]. Therefore, the generic prescribing practice should be encouraged, which allows and eases patients to choose the available cost-effective brand-named medicine; however, the quality of medicines needs to be ensured by concerned regulatory authorities [57].

Interestingly, the average price variation was observed more among the generic medicine items listed in the national and WHO EML and the government-free medicine list. However, none of these characteristics were significantly associated with price variation. This could possibly be due to the small sample size in our study. Also, these categories typically consist of a minimal number of medicines. Generally, the medicines listed in national and WHO EML are mostly old molecules with low or affordable market prices [60]. Government-funded medication programs should incorporate anti-diabetic drugs that are widely used, evidence-based, and recommended by guidelines as safe and effective to ensure access to quality medicines. This measure can help mitigate the potential economic burden that diabetic patients may face in the country. For instance, glimepiride medicine which was recently incorporated into the government’s free medicine list [47, 61]. This is most preferred, practiced, and recommended in relevant guidelines and lists for diabetes treatment in LMICs, including Nepal [50, 54].

Most of the OHMs in the Nepalese market were manufactured within the country (64.7%), which is a good indicator of making the country self-reliant in producing required OHMs. The remaining OHMs were imported from five foreign countries, most from India (31.9%). While the availability of multiple manufacturers’ products provides patients with a wide range of options to make choices, it may also increase medication costs due to increased transportation and marketing costs. This may ultimately cause patients to buy expensive medications as the patients rely solely on doctors’’ suggestions and only take the prescribed brand-named medicines [57, 58]. Therefore, fixing the maximum price in collaboration with the expertise of the pharmaceutical market, prioritising quality over price, could be the appropriate way for patients to reduce the negative impact of price variation. Also, mandating the service of drug and therapeutic committees in hospitals and healthcare facilities may help to manage patients’ medicine access issues with medicine cost, if encountered.

Overall, the wide variation in the price of OHMs observed in the study highlights the possibility of unfair financial burden to diabetic patients who need to take the medications regularly, usually for a lifetime. There may be many contributing factors for such variations, including market influence and infiltration by the pharmaceutical companies, and lack of stringent policies and guidelines from the authorities focusing on the regulations of the market price of medications [24, 57, 62]. Nepal has no clear and transparent medicine pricing policy; even where policies exists, they are not implemented strictly [24, 63]. Shrestha et al. have also revealed that the formulated policies are not effective enough to control the price variation of anticancer medicines in Nepal [63]. The current study presented a picture of the price scenario of OHMs to develop effective drug policies for implementation.

### Strengths and limitations

The strength of this study is that it incorporated almost every OHMs available in Nepal by investigating the pharmacies of tertiary care hospitals of Kathmandu, the online pharmacy platforms and the government medicine database. However, those medicine items that were unavailable and unknown to pharmacy representatives of selected pharmacies, not included in the online pharmacy portal and not updated in the DDA databases were missed to be included. Further, the study did not evaluate the quality aspect of medicine while analysing and discussing the price of medicine. There was a lack of proper system and information to categorize the quality aspect of medicine and, consequently, incorporate that information while analysing the study’s outcome. Additionally, the significance value obtained for the “number of brand-named medicines” and “number of manufacturing countries” variables need to be interpreted cautiously; thus, it is inconclusive due to the limited data for certain categories of these variable.

## Conclusion

Most of the OHMs available in the Nepalese market are self-manufactured. However, the available OHMs, single and fixed-dose combinations, significantly vary in price in Nepal. At the same time, several brand-name medicines and manufacturers provide a possible linkage with price variation but not a definitive relationship. This study presented and discussed the price variation scenario of OHMs in various medicine-related characteristics to develop and implement effective drug policies and programs that can address medication price-related issues to ensure access to OHMs without placing an economic burden on patients. Furthermore, the study recommended exploring the potential impact of price variation on patients’ capacity to afford and adhere to diabetic medicines in the future.

## Supporting information

S1 File Detail price variation table. (XLSX)

S2 File Fixed dose combination price variation table. (XLSX)

## References

[1] World Health Organization. Diabetes. 10 Nov 2021 [cited 29 May 2022]. Available: https://www.who.int/news-room/fact-sheets/detail/diabetes

[2] Saeedi P, Petersohn I, Salpea P, Malanda B, Karuranga S, Unwin N, et al. Global and regional diabetes prevalence estimates for 2019 and projections for 2030 and 2045: Results from the International Diabetes Federation Diabetes Atlas, 9th edition. Diabetes Res Clin Pract. 2019;157: 107843. doi: 10.1016/j.diabres.2019.107843 31518657

[3] Shrestha Shiva Raj Mishra Saruna Ghimire Bishal Gyawali Suresh Mehata N. Burden of Diabetes and Prediabetes in Nepal: A Systematic Review and Meta-Analysis. Diabetes Ther. 11. doi: 10.1007/s13300-020-00884-0 32712902 PMC7434818

[4] Shrestha N, Karki K, Poudyal A, Aryal KK, Mahato NK, Gautam N, et al. Prevalence of diabetes mellitus and associated risk factors in Nepal: findings from a nationwide population-based survey. BMJ Open. 2022;12: 60750. doi: 10.1136/bmjopen-2022-060750 35193925 PMC8867329

[5] Shrestha DB, Budhathoki P, Sedhai YR, Marahatta A, Lamichhane S, Nepal S, et al. Type 2 Diabetes Mellitus in Nepal from 2000 to 2020: A systematic review and meta-analysis. F1000Research. 2021;10: 543. doi: 10.12688/f1000research.53970.1 34621512 PMC8459622

[6] World Health Organization. Diabetes in Nepal. 2016. Available: https://cdn.who.int/media/docs/default-source/country-profiles/diabetes/npl_en.pdf?sfvrsn=6a3e2f17_41&download=true

[7] Lin X, Xu Y, Pan X, Xu J, Ding Y, Sun X, et al. Global, regional, and national burden and trend of diabetes in 195 countries and territories: an analysis from 1990 to 2025. Sci Reports 2020 101. 2020;10: 1–11. doi: 10.1038/s41598-020-71908-9 32901098 PMC7478957

[8] Gyawali B, Ferrario A, van Teijlingen E, Kallestrup P. Challenges in diabetes mellitus type 2 management in Nepal: a literature review. 2016;9. doi: 10.3402/gha.v9.31704 27760677 PMC5071649

[9] Yang JJ, Yu D, Wen W, Saito E, Rahman S, Shu XO, et al. Association of Diabetes with All-Cause and Cause-Specific Mortality in Asia: A Pooled Analysis of More Than 1 Million Participants. JAMA Netw Open. 2019;2: 1–14. doi: 10.1001/jamanetworkopen.2019.2696 31002328 PMC6481439

[10] Ministry of Health and Population. Nepal Burden Of Disease 2019. 2021. Available: http://nhrc.gov.np/wp-content/uploads/2022/02/BoD-Report-Book-includ-Cover-mail-6_compressed.pdf

[11] Shrestha N, Lohani SP, Angdembe MR, Bhattarai K, Bhattarai J. Cost of diabetes mellitus care among patients attending selected outpatient clinics. J Nepal Med Assoc. 2013;52: 343–348. doi: 10.31729/jnma.2114 24362657

[12] Alzaid A, Ladrón de Guevara P, Beillat M, Lehner Martin V, Atanasov P. Burden of disease and costs associated with type 2 diabetes in emerging and established markets: systematic review analyses. 2020. doi: 10.1080/14737167.2020.1782748 32686530

[13] World Heart Organization. WHO | The availability and affordability of selected essential medicines for chronic diseases in six low- and middle-income countries. WHO. 2011. 10.2471/BLT.06.033647 PMC2636320 17546309

[14] Choudhry NK, Denberg TD, Qaseem A. Improving Adherence to Therapy and Clinical Outcomes While Containing Costs: Opportunities From the Greater Use of Generic Medications: Best Practice Advice From the Clinical Guidelines Committee of the American College of Physicians. Ann Intern Med. 2016;164: 41. doi: 10.7326/M14-2427 26594818

[15] McGuire M, Iuga. Adherence and health care costs. Risk Manag Healthc Policy. 2014;7: 35. doi: 10.2147/RMHP.S19801 24591853 PMC3934668

[16] Ministry of Health and Population Government of Nepal. Nepal National Health Accounts 2012/13 to 2015/16. 2018. Available: www.searo.who.int/entity/nepal/documents/nepal_nha_2012_13_2015_16_mohp_june_2018.pdf

[17] Ide N, LoGerfo JP, Karmacharya B. Barriers and facilitators of diabetes services in Nepal: A qualitative evaluation. Health Policy Plan. 2018;33: 474–482. doi: 10.1093/heapol/czy011 29447397

[18] Sah BK, Basyal D, Gaire A. Medication Non-Adherence among Type-II Diabetes Mellitus Out-Patients Attending at Tertiary Care Hospital, Nepal. Clin Pharmacol Biopharm. 2021. doi: 10.4172/2167-065X.1000236

[19] Shrestha B. Cost analysis study of oral hypoglycemic agents available in Nepalese market. Med J Shree Birendra Hosp. 2015;13: 6–9. doi: 10.3126/mjsbh.v13i2.13107

[20] Poudel RS, Poudel BK, Shrestha S, Piryani RM. Variation in prices of medicines used for the long-term management of non-communicable diseases available in the pharmacy of a tertiary care hospital of Nepal. J Pharm Heal Serv Res. 2018;9: 293–296. doi: 10.1111/JPHS.12237

[21] Shrestha R, Ghale A, Chapagain BR, Gyawali M, Acharya T. Survey on the availability, price and affordability of selected essential medicines for non-communicable diseases in community pharmacies of Kathmandu valley. SAGE Open Med. 2017;5: 205031211773869. doi: 10.1177/2050312117738691 29201366 PMC5697585

[22] Department of Drug Administration. List of importers and companies from which they import medicines. 2018 [cited 2 Jun 2022]. Available: https://www.dda.gov.np/content/list-of-importers-and-companies-from-which-they-import-medicines-till-4-10-2018-on-dams

[23] Department of Drug Administration. List of Domestic industries. 2021 [cited 2 Jun 2022]. Available: https://www.dda.gov.np/content/list-of-domestic-industries-listed-in-dams-till-14-06-2021

[24] USAID MTaPS Program. Strengthening Pharmaceutical Regulatory Systems in the Asian Region. 2023. Available: https://www.mtapsprogram.org/our-resources/strengthening-pharmaceutical-regulatory-systems-in-the-asian-region/

[25] Babar Z-U-D, Dulal S, Dhakal NP, Upadhyaya MK, Trap B. Developing Nepal’s medicines pricing policy: evidence synthesis and stakeholders’ consultation. J Pharm Policy Pract. 2024;17: 2346222. doi: 10.1080/20523211.2024.2346222 38690551 PMC11060005

[26] Government of Nepal. MRP of medicines. In: Department of Drug Administration [Internet]. 2018 [cited 8 Oct 2022]. Available: https://www.dda.gov.np/content/mrp-of-medicines

[27] Ministry of Health and Population Nepal. List of Essential Medicines for Basic Health Services in Nepal. 2020 [cited 28 Aug 2022]. Available: https://publichealthupdate.com/list-of-free-essential-drugs-for-health-institutions-nepal/

[28] Nepal Government. Health Insurance Board. [cited 31 May 2022]. Available: https://hib.gov.np/en/

[29] Aryal A, Dahal A, Shrestha R. Study on drug use pattern in primary healthcare centers of Kathmandu valley. SAGE Open Med. 2020;8: 205031212092643. doi: 10.1177/2050312120926437 32499916 PMC7243404

[30] Nepal Health Research Council. Quality of Essential Medicines in Public Health Care Facilities of Nepal. 2019. Available: http://nhrc.gov.np/wp-content/uploads/2020/08/Drug-report.pdf

[31] Sharma P, Kumar Yadav D, Shrestha N, Ghimire P. Dropout Analysis of a National Social Health Insurance Program at Pokhara Metropolitan City, Kaski, Nepal. Int J Heal Policy Manag. 2021;2021: 1–13. doi: 10.34172/ijhpm.2021.171 35042322 PMC9818104

[32] Ranabhat CL, Subedi R, Karn S. Status and determinants of enrollment and dropout of health insurance in Nepal: An explorative study. Cost Eff Resour Alloc. 2020;18: 1–13. doi: 10.1186/S12962-020-00227-7/TABLES/5 33013204 PMC7528465

[33] Matuszewski K, Kapusnik-Uner J, Man M, Pardini R, Suko J. Variation in Generic Drug Manufacturers’ Product Characteristics. P T. 2018;43: 485–504. Available: http://www.ncbi.nlm.nih.gov/pubmed/30100689 30100689 PMC6065490

[34] ZG O. Regulatory Analysis of Mark-up Structure in Medicine Prices by the Pharmaceutical Industry in South Africa. J Pharm Care Heal Syst. 2019;6: 1–7. doi: 10.35248/2376-0419.19.6.203

[35] Chaudhuri S. Pharmaceutical Prices in India. Pharmaceutical Prices in the 21st Century. Cham: Springer International Publishing; 2015. pp. 113–130. doi: 10.1007/978-3-319-12169-7_7

[36] Burapadaja S, Kawasaki N, Kittipongpatana O, Ogata F. Study on variations in price of prescription medicines in Thailand. Yakugaku Zasshi. 2007;127: 515–26. doi: 10.1248/yakushi.127.515 17329937

[37] Khanal S, Veerman L, Ewen M, Nissen L, Hollingworth S. Availability, Price, and Affordability of Essential Medicines to Manage Noncommunicable Diseases: A National Survey From Nepal. Inq J Heal Care Organ Provision, Financ. 2019;56. doi: 10.1177/0046958019887572 31823665 PMC6906349

[38] Devkota A, Paudel A, Koirala B, Baral D, Gautam S, Kumar Sharma S, et al. Price Variation and Availability of Free Medicine for Non-communicable Diseases. Jnhrc. 2018;16: 118–123. doi: 10.3126/jnhrc.v16i2.20295 29983422

[39] Upadhyay N. Imprecise drug pricing for metformin in Nepalese Market. World J Pharm Res. 2015;4. Available: https://wjpr.s3.ap-south-1.amazonaws.com/article_issue/1438333958.pdf

[40] Annapurna Pharmacy. [cited 23 Feb 2023]. Available: http://annapurnapharmacy.com/

[41] ePharmacy. [cited 23 Feb 2023]. Available: https://www.epharmacy.com.np/

[42] Department of Drug Administration. Registered Drug Info. [cited 31 Oct 2022]. Available: http://dams.dda.gov.np/manLogin/publicInfo

[43] Feingold KR. Oral and Injectable (Non-Insulin) Pharmacological Agents for the Treatment of Type 2 Diabetes. Endotext. 2021 [cited 2 Jun 2022]. Available: https://www.ncbi.nlm.nih.gov/books/NBK279141/25905364

[44] Department of Drug Adhministration. National List of Essential Medicines Nepal. 2021. Available: https://scorecard.prb.org/wp-content/uploads/2022/03/National-List-of-Essential-Medicines-Nepal-2021.pdf

[45] World Health Organization. WHO model list of essential medicines - 22nd list, 2021. Tech Doc. 2021; 2021. Available: https://www.who.int/publications/i/item/WHO-MHP-HPS-EML-2021.02

[46] Government of Nepal. List and Price of Medicines and Facilities Available From the Health Insurance Nepal. Available: https://pharmainfonepal.com/list-and-price-of-medicines-and-facilities-available-from-the-health-insurance-nepal/

[47] Bista N. List of Free Medicine in Nepal. In: Pharma Info Nepal [Internet]. 2022. Available: https://pharmainfonepal.com/list-of-free-medicine-in-nepal/

[48] Dinesh KU, Subish P, Pranaya M, Shankar PR, Anil SK, Durga B. Pattern of potential drug-drug interactions in diabetic out-patients in a tertiary care teaching hospital in Nepal. Med J Malaysia. 2007;62: 294–8. Available: http://www.ncbi.nlm.nih.gov/pubmed/18551932 18551932

[49] Dhakal Saroj, Shakya Shrijana, Shree Krishna Sharma. Cost-Effectiveness Analysis of Oral Hypoglycemics for Type-2 Diabetes Mellitus at a Tertiary Care Hospital, Nepal. J Pharm Pharmacol. 2019;7: 546–556. doi: 10.17265/2328-2150/2019.10.004

[50] Dahal P, Maharjan L, Dahal B, Gupta K. Assessment of Prescription Patterns in Hypertensive and Diabetic Patients Visiting Private Tertiary Care Hospital of Dharan Municipality, Nepal. Sunsari Tech Coll J. 2016;2: 44–47. doi: 10.3126/stcj.v2i1.14798

[51] Nazrina S, Maruf A Al, Rahman W, Khan FZ. Prescribing Pattern of Anti-Diabetic Drugs for Type 2 Diabetic Patients in a Tertiary Care Hospital. J Armed Forces Med Coll Bangladesh. 2020;14: 139–143. doi: 10.3329/jafmc.v14i2.45894

[52] Tanwar S, Acharya A, Hasan N. Assessment of drug utilization pattern of antidiabetic drugs in type-2 diabetes outpatient of a tertiary care teaching hospital western Rajasthan. Int J Basic Clin Pharmacol. 2021;10: 368. doi: 10.18203/2319-2003.ijbcp20211017

[53] Dhongdi SR, Siddiqui RA. Polypharmacy and cost analysis in patients suffering from type 2 diabetes mellitus with associated comorbidities. Int J Basic Clin Pharmacol. 2020;9: 929. doi: 10.18203/2319-2003.ijbcp20202184

[54] Mohan V, Saboo B, Khader J, Modi KD, Jindal S, Wangnoo SK, et al. Position of Sulfonylureas in the Current ERA: Review of National and International Guidelines. Clin Med Insights Endocrinol Diabetes. 2022;15: 117955142210746. doi: 10.1177/11795514221074663 35185350 PMC8854230

[55] Gupta KS, Pardeshi ML, Hiray RS. Cost variation analysis of commonly prescribed anti-diabetic drugs available in Indian market: a pharmaco-economic study. Int J Basic Clin Pharmacol. 2021;11: 47. doi: 10.18203/2319-2003.IJBCP20214888

[56] Shrestha R, Gurung S. An evaluation of fixed dose combinations and it’s practices at medicine department of tertiary care hospital, Nepal. Int J Sci Reports. 2020;6: 67. doi: 10.18203/issn.2454-2156.IntJSciRep20200197

[57] Shrestha R, Shrestha S, Sapkota B, Thapa S, Ansari M, Khatiwada AP, et al. Generic Medicine and Generic Prescribing in Nepal: An Implication for Policymakers. J Multidiscip Healthc. 2022;Volume 15: 365–373. doi: 10.2147/JMDH.S348282 35237042 PMC8884710

[58] Ansari M, Humagain B, Hassali MA. Generic medicines utilizations and generic prescribing in Nepal: A reflection of current scenario and possible solutions. Research in Social and Administrative Pharmacy. Elsevier Inc.; 2017. pp. 658–659. doi: 10.1016/j.sapharm.2017.01.004 28162993

[59] Shrestha R, Prajapati S. Assessment of prescription pattern and prescription error in outpatient Department at Tertiary Care District Hospital, Central Nepal. J Pharm Policy Pract. 2019;12: 16. doi: 10.1186/s40545-019-0177-y 31321037 PMC6617589

[60] Kar SS, Pradhan HS, Mohanta GP. Concept of essential medicines and rational use in public health. Indian J Community Med. 2010;35: 10. doi: 10.4103/0970-0218.62546 20606912 PMC2888334

[61] Nepal G of. List of Free Essential Medicines. 2018. Available: http://dohs.gov.np/wp-content/uploads/2018/08/Free_Drugs_List.pdf

[62] Jha N, Sapkota Y, Shankar PR. Critical evaluation of drug advertisements in a medical college in Lalitpur, Nepal. J Multidiscip Healthc. 2020;13: 717–725. doi: 10.2147/JMDH.S259708 32801734 PMC7406374

[63] Shrestha S, Poudel RS, KC B, Poudel BK, Sapkota B, Sharma S, et al. Price variation among different brands of anticancer medicines available in hospital pharmacies of Nepal. J Pharm Policy Pract. 2020;13: 6. doi: 10.1186/s40545-020-0203-0 32266073 PMC7118972

